# Synthesis and Evaluation of ^177^Lu-DOTA-PD-L1-i and ^225^Ac-HEHA-PD-L1-i as Potential Radiopharmaceuticals for Tumor Microenvironment-Targeted Radiotherapy

**DOI:** 10.3390/ijms241512382

**Published:** 2023-08-03

**Authors:** Myrna Luna-Gutiérrez, Pedro Cruz-Nova, Nallely Jiménez-Mancilla, Rigoberto Oros-Pantoja, Nancy Lara-Almazán, Clara Santos-Cuevas, Erika Azorín-Vega, Blanca Ocampo-García, Guillermina Ferro-Flores

**Affiliations:** 1 Department of Radioactive Materials, Instituto Nacional de Investigaciones Nucleares, Ocoyoacac 52750, Mexicoerica.azorin@inin.gob.mx (E.A.-V.); 2 Cátedras, CONACyT, Instituto Nacional de Investigaciones Nucleares, Ocoyoacac 52750, Mexico; 3 Faculty of Medicine, Universidad Autónoma del Estado de México, Toluca 50180, Mexico

**Keywords:** PD-L1 radioinhibitors, actinium-225, lutetium-177, targeted radiotherapy

## Abstract

Current cancer therapies focus on reducing immunosuppression and remodeling the tumor microenvironment to inhibit metastasis, cancer progression, and therapeutic resistance. Programmed death receptor 1 (PD-1) is expressed on immune T cells and is one of the so-called checkpoint proteins that can suppress or stop the immune response. To evade the immune system, cancer cells overexpress a PD-1 inhibitor protein (PD-L1), which binds to the surface of T cells to activate signaling pathways that induce immune suppression. This research aimed to synthesize PD-L1 inhibitory peptides (PD-L1-i) labeled with lutetium-177 (^177^Lu-DOTA-PD-L1-i) and actinium-225 (^225^Ac-HEHA-PD-L1-i) and to preclinically evaluate their potential as radiopharmaceuticals for targeted radiotherapy at the tumor microenvironment level. Using PD-L1-i peptide as starting material, conjugation with HEHA-benzene-SCN and DOTA-benzene-SCN was performed to yield DOTA-PD-L1-i and HEHA-PD-L1-I, which were characterized by FT-IR, UV-vis spectroscopy, and HPLC. After labeling the conjugates with ^225^Ac and ^177^Lu, cellular uptake in HCC827 cancer cells (PD-L1 positive), conjugate specificity evaluation by immunofluorescence, radiotracer effect on cell viability, biodistribution, biokinetics, and assessment of radiation absorbed dose in mice with in duced lung micrometastases were performed. ^225^Ac-HEHA-PD-L1-i and ^177^Lu-DOTA-PD-L1-i, obtained with radiochemical purities of 95 ± 3% and 98.5 ± 0.5%, respectively, showed in vitro and in vivo specific recognition for the PD-L1 protein in lung cancer cells and high uptake in HCC287 lung micrometastases (>30% ID). The biokinetic profiles of ^177^Lu-DOTA-PD-L1-i and ^225^Ac-DOTA-PD-L1-i showed rapid blood clearance with renal and hepatobiliary elimination and no accumulation in normal tissues. ^225^Ac-DOTA-PD-L1-i produced a radiation dose of 5.15 mGy/MBq to lung micrometastases. In the case of ^177^Lu-DOTA-PD-L1-i, the radiation dose delivered to the lung micrometastases was ten times (43 mGy/MBq) that delivered to the kidneys (4.20 mGy/MBq) and fifty times that delivered to the liver (0.85 mGy/MBq). Therefore, the radiotherapeutic PD-L1-i ligands of ^225^Ac and ^177^Lu developed in this research could be combined with immunotherapy to enhance the therapeutic effect in various types of cancer.

## 1. Introduction

Tumors are pathological tissues that comprise cancer cells and the tumor microenvironment (TME). The TME includes endothelial and immune cells communicating in a dynamic network through messenger molecules and acellular factors, mainly chemokines, inflammatory enzymes, growth factors, cytokines, and extracellular matrix components [1]. The function of immune cells and messenger molecules associated with the TME is a relevant topic in the research field of cancer biology.

Therefore, current cancer therapies converge on inhibiting cancer progression and therapeutic resistance by reducing immunosuppression and remodeling the TME [2,3]. In this context, previous research on the development of imaging radioligands targeting proteins of the tumor microenvironment, such as nanobodies against proteins of the extracellular matrix [4], radioligands to target the chemokine receptor-4 (CXCR-4) [5,6,7], fibroblast activation protein (FAP) radioinhibitors [8,9,10,11,12], and radiotracers directed to integrins and the prostate-specific membrane antigen (PSMA) expressed in the tumor neovasculature [13,14,15,16,17,18,19], among others, have proven to be helpful in detecting spatial and temporal changes in the tumor microenvironment phenotype of different cancer entities, which have been essential in defining the treatment of patients according to the expression or suppression of proteins involved in the disease. In particular, the immunotherapeutic pathway has been exploited with various agents, such as monoclonal antibodies, radioimmunoconjugates, and nano-immune systems [20,21].

Immune checkpoint inhibitor (ICI) therapy is an important treatment option for various cancers [22,23]. Among the targeted immune checkpoints, programmed death receptor 1 (PD-1) and its ligand (PD-L1) are the key molecular targets for ICI therapy. Expressed on immune T cells, PD-1 is one of the checkpoint molecules that can suppress or stop immune responses [22]. Therefore, to evade the immune system, cancer cells overexpress a PD-1 inhibitor protein (PD-L1) that binds to the surface of T cells to activate signaling pathways that induce immune suppression. Thus, PD-L1 is a reproducible biomarker that allows therapeutic decisions to be made and the success of the treatment to be monitored. PD-L1 gene expression imaging, which is highly dynamic, can be monitored in a highly specific manner by SPECT/CT and PET/CT imaging techniques, which are used to determine whether a patient is a candidate for immunotherapeutic treatment with anti-PD-L1 or anti-PD-1 antibodies [24,25,26,27]. However, PD-L1 inhibitor radiotherapeutic peptides have not yet been investigated. Lutetium-177 is a β-emitting radionuclide with a mean tissue penetration of 0.67 mm (maximum energy: 497 keV), and actinium-225 is an alpha-emitting radionuclide with a maximum tissue penetration of a few cell diameters (emission of four high-energy alpha particles with an energy of 6 MeV). Therefore, both radionuclides have sufficient energy and tissue penetration to kill target tumor cells with limited effect on adjacent healthy cells.

This research aimed to synthesize lutetium-177- and actinium-225-labeled PD-L1 inhibitory peptides (^177^Lu-DOTA-PD-L1-i and ^225^Ac-HEHA-PD-L1-i) and to preclinically evaluate their potential as radiopharmaceuticals for targeted radiotherapy of various cancers at the tumor microenvironment level.

## 2. Results and Discussion

### 2.1. Synthesis and Chemical Characterization of DOTA-PD-L1-i and HEHA-PD-L1-i

**Figure A1:**
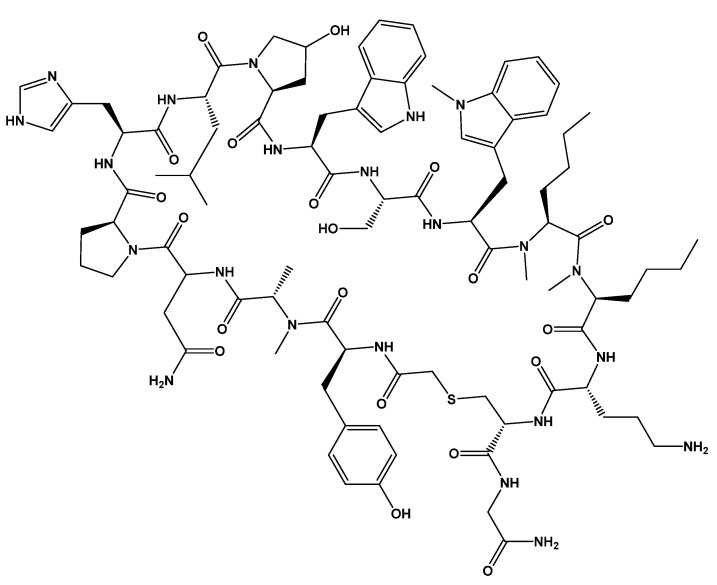
Schematic chemical structure of the PD-L1-i ligand.

**Figure A2:**
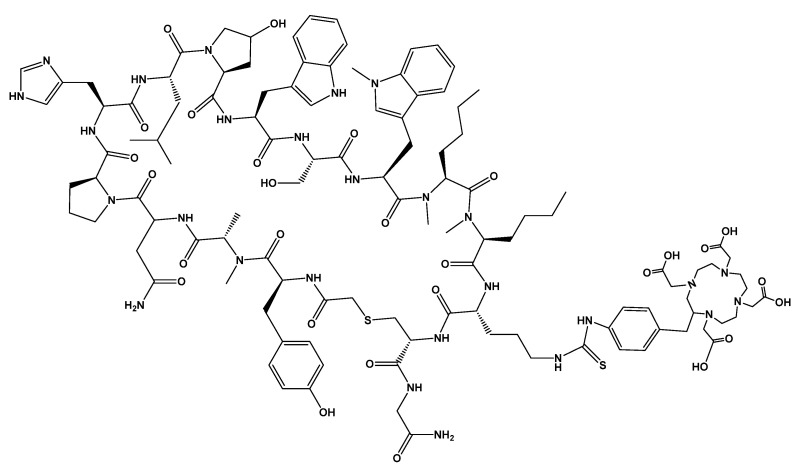
Schematic chemical structure of the DOTA-benzene-SCN-PD-L1-i (DOTA-PD-L1-i) ligand.

**Figure A3:**
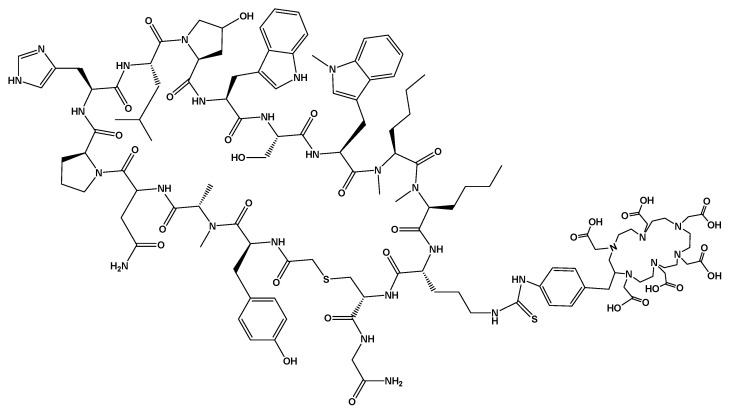
Schematic chemical structure of the HEHA-benzene-SCN-PD-L1-i (HEHA-PD-L1-i) ligand.

The schematic chemical structures of PD-L1-i, DOTA-PD-L1-i, and HEHA-PD-L1-i are shown in Appendix A (Figure A1, Figure A2 and Figure A3).

**Figure 1:**
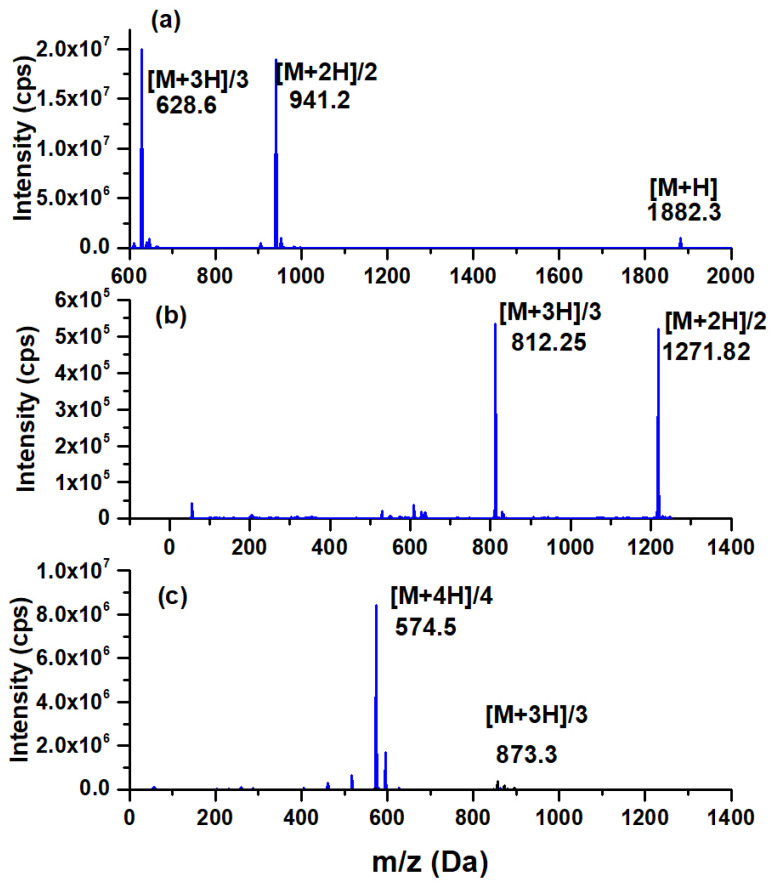
Mass spectrum of the (**a**) PD-L1-i peptide: cyclo(Ac-Tyr-NMeAla-Asn-Pro-His-Leu-Hyp-Trp-Ser-Trp(Me)-NMeNle-NMeNle-Orn-Cys-Gly-NH_2_ synthesized as previously reported [28], (**b**) DOTA-PD-L1-i, and (**c**) HEHA-PD-L1-i.

The PD-L1-i mass spectrum showed *m*/*z* of 1882.3 (calcd. 1881) [M + H], *m*/*z* 941.2 (calcd. 1881) [M + 2H]/2, and *m*/*z* 628.6 (calcd. 1881) [M + 3H]/3 (Figure 1). From the PD-L1-i compound as row material, the conjugation with HEHA-benzene-SCN and DOTA-benzene-SCN was performed to obtain DOTA-PD-L1-i (*m*/*z* 1271.8, calcd. 2541.6, [M + 2H]/2) and HEHA-PD-L1-i (*m*/*z* 873.3, calcd. 2633.9 minus –OH in HEHA, [M + 3H]/3; at *m*/*z* 867, two –OH were lost and at *m*/*z* 574, –CH3–COOH was lost in HEHA) (Figure 1). The PD-L1 inhibitor ligands (PD-L1-i, DOTA-PD-L1-i, and HEHA-PD-L1-i) were also characterized using FT-IR, UV-vis spectroscopy, and HPLC, as described below.

**Figure 2:**
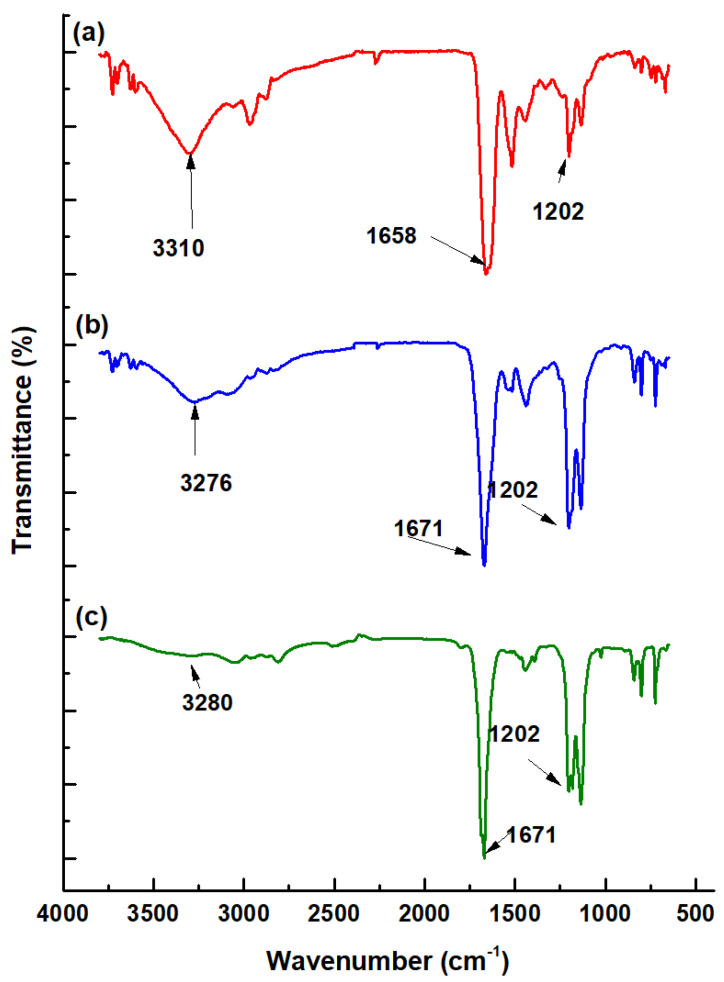
FT-IR spectra: (**a**) PD-L1-i peptide; (**b**) DOTA-PD-L1-i; (**c**) HEHA-PD-L1-i.

The IR analysis of the PD-L1 inhibitor conjugates showed that the band at 1658 cm^−1^ of PD-L1-i, associated with the –C=O stretching of carbonyl bonds (–*CO*–NH–), decreased in energy to 1671 cm^−1^ in both DOTA-PD-L1-i and HEHA-PD-L1-i as a result of the conjugation reaction (–HN–*CS*–NH-benzene-DOTA/-HEHA), which produced a decrease in the absorption frequency, as dipole moments in the cyclic peptide ring were reduced (Figure 2). On the other hand, the band at 3310 cm^−1^ of PD-L1-I, associated with the primary amine of ornithine (–NH_2_), increased in energy to 3276 cm^−1^ and 3280 cm^−1^ in the DOTA-PD-L1-i and HEHA-PD-L1-i conjugates, respectively, which is characteristic of –NH– bond formation (–*HN*–CS–). Further evidence for the conjugation process was the substantial increase in the band’s intensity at 1202 cm^−1^ associated with a rise in the –C–O– stretching from DOTA and HEHA carboxylic acids (O=*CO*–H) (Figure 2).

**Figure 3:**
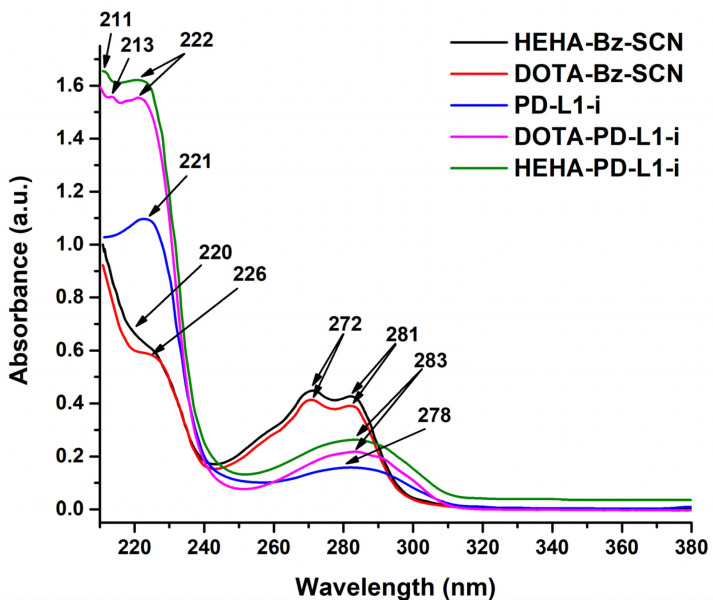
UV-vis spectra of PD-L1-i peptide, DOTA-PD-L1-I, and HEHA-PD-L1-i.

The UV-vis spectra of the PD-L1-i conjugates show several shoulders and peaks from 211 nm to 226 nm, which are assigned to the n→σ* electronic transitions from sulfur as a heteroatom (-H_2_C–S–CH_2_^−^), π→π* electronic transitions from double bonds within amino acid carbonyls, and –C=O from carboxylic acids. The band at 283 nm is associated with π→π* and n→π* electronic transitions (–C=C– and –C=N– groups) of the peptide side chain rings (e.g., imidazole ring) (Figure 3B). The single broad and well-defined band at 283 nm of DOTA-PD-L1-i and HEHA-PD-L1-i appears different from that of the precursors (278 nm from the PD-L1-i ligand and the 272 nm and 281 nm doublet from the DOTA-/HEHA-Benzene-SCN precursors), indicating new chemical compound formation (PD-L1-i conjugates) (Figure 3).

### 2.2. Radiolabeling of PD-L1-i Ligands with ^177^Lu and ^225^Ac

**Figure 4:**
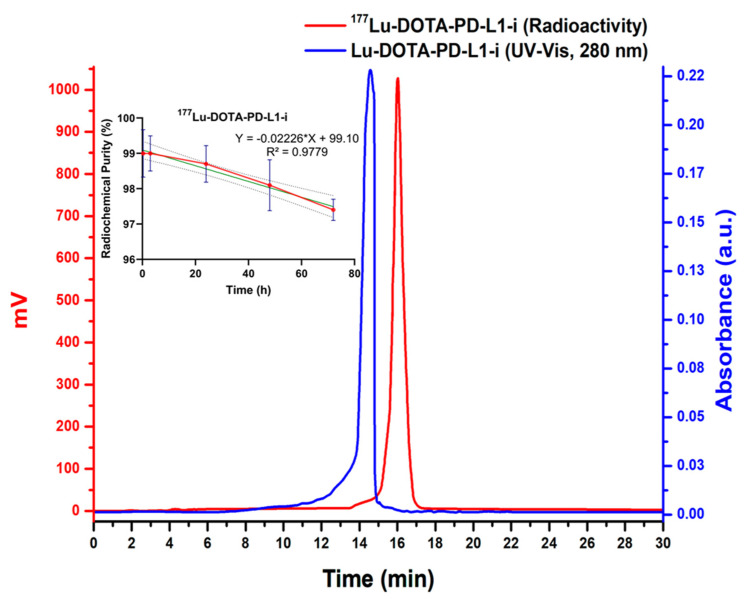
Reversed-phase UV-vis/HPLC, 0.1 mg/mL of Lu-DOTA-PD-L1-i in aqueous solution, injection of 20 µL, and reversed-phase radio-HPLC chromatogram of ^177^Lu-DOTA-PD-L1-i in water (ascorbic acid/sodium acetate), 37 MBq/mL, injection of 20 µL (inset: ^177^Lu-DOTA-PD-L1-i stability in water).

**Figure 5:**
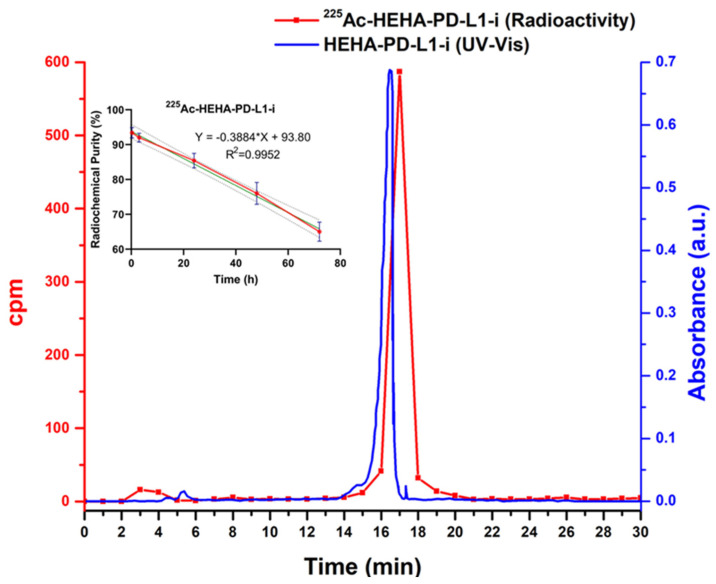
Reversed-phase UV-vis (280 nm)/HPLC, 0.2 mg/mL of HEHA-PD-L1-i in aqueous solution, injection of 20 µL, and reversed-phase HPLC separation of ^225^Ac-HEHA-PD-L1-i in water (ascorbic acid/sodium acetate), 1.85 MBq/mL, injection of 20 µL (graph of collected fractions) (inset: ^225^Ac-HEHA-PD-L1-i stability in water).

The radiopharmaceuticals ^177^Lu-DOTA-PD-L1-i and ^225^Ac-HEHA-PD-L1-i were initially obtained with radiochemical purities of 98.5 ± 0.5% (without further purification) and 83 ± 2% (95 ± 3% after purification using Sep-Pak C18 cartridges), respectively, as determined by radio-HPLC (C-18 reversed phase) (Figure 4 and Figure 5). However, to assess the radiochemical purity of ^225^Ac-HEHA-PD-L1-i, fractions of 1 mL eluted from the column had to be collected and counted in a NaI(Tl) detector (Figure 5). Retention times for ^177^LuCl_3_ and ^225^AcCl_3_ were between 2.5 and 4.5 min and 16 to 18 min for ^177^Lu-DOTA-PD-L1-i and ^225^Ac-HEHA-PD-L1-i. It is important to note that chromatograms are usually achieved with radiometric and UV-vis detectors. After injecting the radiotracer into the HPLC system, it was first identified using the UV-vis instrument, and after 0.8–1.0 min (0.8–1.0 mL, 1 mL/min), it was detected with the radiometric detector. An agreement of retention times between the peak observed in the UV-vis chromatogram (PD-L1-i conjugate) and the radio-chromatogram is usually considered evidence of the chemical identity of the radiopharmaceutical (Figure 4) [29].

### 2.3. In Vitro Evaluation

#### 2.3.1. Stability

PD-L1 inhibitor radioconjugates showed no differences regarding stability as a function of the dilution medium. ^177^Lu-DOTA-PD-L1-i remained stable in water (injectable-grade water/ascorbic acid/sodium acetate) and human serum with radiochemical purities greater than 97.5 ± 0.5% after 72 h of labeling (inset: Figure 4). In contrast, the radiochemical purity of ^225^Ac-HEHA-PD-L1-i decreased to 64 ± 4% in both water and human serum 72 h after purification, at a rate of approximately 0.388%/h (slope = −0.388) (inset: Figure 5). It is important to mention that, although it was not the aim of this study, we also examined the preparation of ^225^Ac-DOTA-PD-L1-i, which showed a radiochemical purity of 9 ± 3% in water after 72 h of labeling (1.149%/h rate of decrease). It is well documented that the main disadvantage of ^225^Ac complexes is the recoil energy of the nucleus during decay, which induces the breaking of the bond with the chelator [30]. In agreement with previous research, the use of HEHA instead of DOTA improves the stability of the coordination complex [31,32]. Nevertheless, the recoil energy of ^225^Ac and its progeny (four alpha particles) will inevitably produce, to a greater or lesser extent, time-unstable ^225^Ac complexes [30,33].

#### 2.3.2. Cellular Uptake Assay

**Figure 6:**
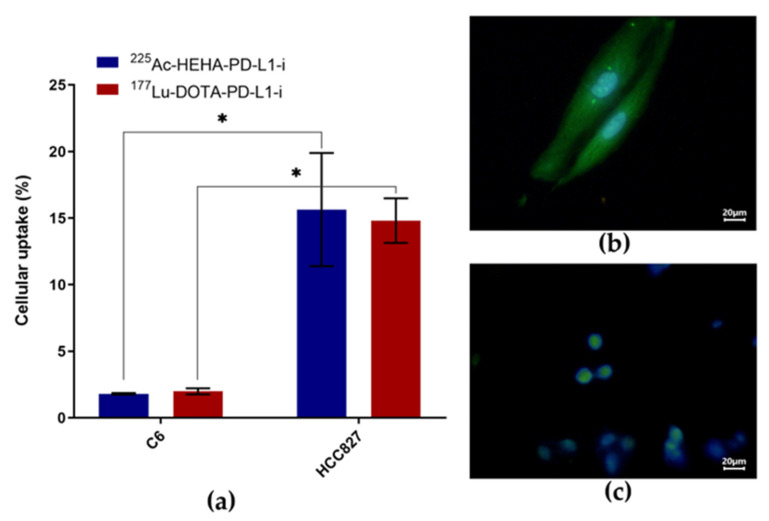
(**a**) In vitro ^177^Lu-DOTA-PD-L1-i and ^225^Ac-HEHA-PD-L1-i uptake in C6 mouse glioma cancer cells (PD-L1 negative control) and HCC827 human lung cancer cells (PD-L1 positive control) (n = 6). * Significant difference, *p* < 0.05; Student’s *t*-test. Microscopic fields (40x) of immunofluorescent staining for PD-L1 in (**b**) HCC827 (PD-L1+) cells (merged images: PD-L1 stained green with anti-PD-L1 and nuclei stained blue with DAPI) and (**c**) C6 cells negative for PD-L1 expression (merged images: anti-PD-L1 green and DAPI blue for nuclei staining).

**Figure 7:**
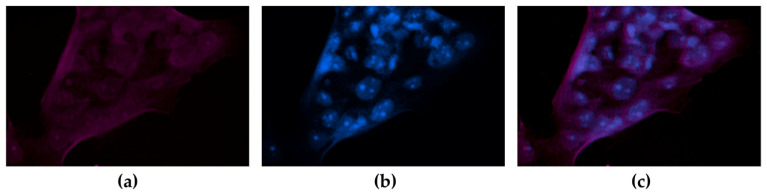
Microscopic field (40×) of immunofluorescent staining for PD-L1 with the (**a**) Cy7-labeled PD-L1-i in HCC827 (PD-L1+) cells; (**b**) DAPI staining; (**c**) merged image: PD-L1 stained red with Cy7-PD-L1-i and nuclei stained blue with DAPI.

HCC827 cells (PD-L1 positive control) were found to uptake approximately fourteen times more ^177^Lu-DOTA-PD-L1-i and ^225^Ac-HEHA-PD-L1-i than C6 cells (PD-L1 negative control) (Figure 6a). The expression of PD-L1 in HCC827 cells (Figure 6b) and the lack of PD-L1 expression in C6 cells (Figure 6c) were confirmed by immunofluorescent microscopy, thus ensuring that the radiotracer uptake is specific and associated with the presence of PD-L1 in HCC827 cells. It was also proven by immunofluorescent microscopy that the PD-L1-i ligand, used in this research for ^177^Lu and ^225^Ac labeling, is recognized by the PD-L1 protein expressed in HCC827 cells (Figure 7).

#### 2.3.3. Cell Viability Assays

**Figure 8:**
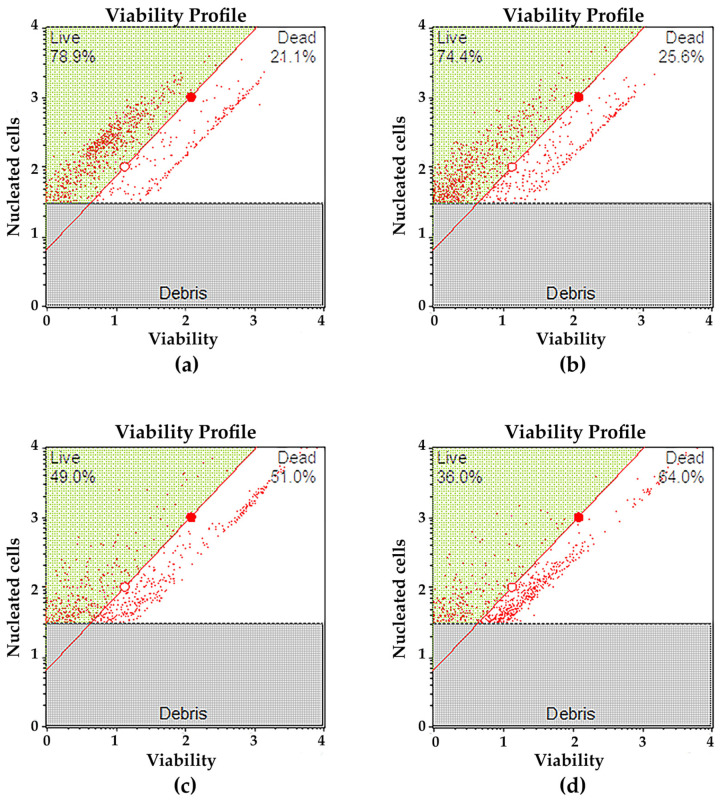
Representative histograms of the HCC827 cell viability assay obtained using flow cytometry: (**a**) control (no treatment): basal viability of the cell population (78.9% of live cells); (**b**) viability after treatment with PD-L1-i (0.1 µg) for 5-h (74.4% of live cells); (**c**) viability after treatment (acute dose of 15 Gy/5 h) with ^177^Lu-DOTA-PD-L1-i (49.0% of live cells; the net decrease in alive cells regarding the control group was 30%); (**d**) viability after treatment (acute dose of 5 Gy/5 h) with ^225^Ac-HEHA-PD-L1-i (36.0% of live cells; the net decrease in alive cells regarding the control group was 43%).

A decrease in the viability of HCC827 cells was observed depending on the target-specific radiotherapy mediated by the PD-L1 recognition (Figure 8). Cells treated with an acute radiation dose of 15 Gy using ^177^Lu-DOTA-PD-L1-i (cells incubated for 5 h with ^177^Lu-DOTA-PD-L1-i; 1.4 MBq/20,000) showed a net decrease viability of 31.42 ± 2.01%, with a statistically significant difference (*p* < 0.05, Student’s *t*-test) compared to the group treated with PD-L1-i (decrease viability of 4 ± 1%; 0.1 µg/20,000 cells) (Figure 8b,c). The viability of cells treated with 5 Gy of ^225^Ac-HEHA-PD-L1-i (cells incubated for 5 h with ^225^Ac-HEHA-PD-L1-i; 0.37 kBq/20,000 cells) had a net decrease viability of 42.54 ± 3.87% vs. 4 ± 1% of those treated with PD-L1-i (Figure 8b,d). The reason why we used a three times lower radiation dose for ^225^Ac-HEHA-PD-L1-i (5 Gy) than for ^177^Lu-DOTA-PD-L1-i (15 Gy) was the consideration of the high number of short path ionizations generated by alpha particle emitters (high LET) damaging DNA structures. This effect, known as RBE (relative biological efficiency), explains why actinium-225 affected cell viability more than lutetium-177, even at a lower radiation dose [34].

### 2.4. In Vivo Evaluation

#### 2.4.1. Biodistribution and Biokinetic Models

**Figure 9:**
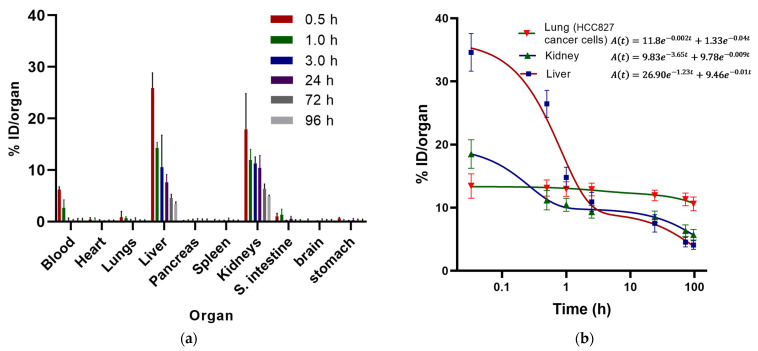
(**a**) Biodistribution profile in healthy mice of ^177^Lu-DOTA-PD-L1-i; (**b**) biokinetic models of ^177^Lu-DOTA-PD-L1-i in the three target tissues: lung (invaded by HCC827 lung cancer cells), liver, and kidney.

**Figure 10:**
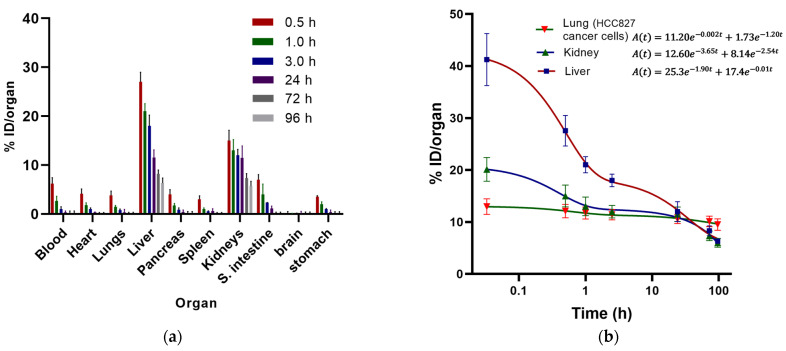
(**a**) Biodistribution profile in healthy mice of ^225^Ac-HEHA-PD-L1-i; (**b**) biokinetic models of ^225^Ac-HEHA-PD-L1-I in the three target tissues: lung (invaded by HCC827 lung cancer cells), liver, and kidney.

In healthy mice, the ^177^Lu-DOTA-PD-L1-i and ^225^Ac-DOTA-PD-L1-i biodistribution profiles showed rapid blood clearance with renal and hepatobiliary elimination and no accumulation in normal tissues. (Figure 9a and Figure 10a).

According to the biokinetic model of ^177^Lu-DOTA-PD-L1-i obtained in mice intravenously injected with HCC827 lung cancer cells (PD-L1+) (Figure 9b), seventy-four percent of the activity taken up in the liver was cleared rapidly (λ = 1.23), with a half-life of 0.56 h (t_1/2_ = ln2/λ = 0.56 h) associated with the hepatobiliary elimination, while twenty-six percent was cleared slowly (λ = 0.01; t_1/2_ = 69 h), possibly related to the fraction retained in the reticuloendothelial system. The short half-life time observed in the biokinetic model of kidneys (t_1/2_ = ln2/3.65 = 0.19 h) can be correlated with renal elimination and reabsorption in the proximal tubule, whereas the slow elimination slope (t_1/2_ = ln2/0.009 = 77 h) would be related to the breakdown of the peptide fragment, where ^177^Lu is retained for a longer time [35]. The most extended ^177^Lu-DOTA-PD-L1-i retention time was observed in lungs invaded with HCC827 cancer cells. Ninety percent of the lung uptake activity had a half-life of 346.5 h, and only ten percent had a half-life of 17.3 h (Figure 9b); consequently, the absorbed radiation dose delivered to the lungs was ten times that given to the kidneys and fifty times than that to the liver (Table 1).

In the case of the ^225^Ac-HEHA-PD-L1-i radiocomplex, the biodistribution profile during the first hours after injection showed higher uptake of radioactivity in most of the organs examined and a significantly higher hepatic uptake and retention (17.4% of the injected dose was eliminated with a half-life of 69.3 h) than in the case of the ^177^Lu-DOTA-PD-L1-i complex, which may be related to the presence of Ac^3+^ and its progeny released from the radiocomplex, initially distributed in healthy organs and subsequently retained in the liver and, to a lesser extent, in the kidney (12.6% of the injected dose was eliminated with a half-life of 0.19 h and 8.14% with a half-life of 0.27 h) (Figure 10). Therefore, the absorbed radiation dose delivered to the lungs was almost equal to that of the kidneys and only 3.5 times that given to the liver (Table 1). Although biodistribution was performed for dosimetric purposes (only %ID in whole organs was calculated), the Ac-225 activity present in bone was also measured and was not significant (0.3 ± 0.2% ID/g).

#### 2.4.2. Radioisotopic Imaging

**Figure 11:**
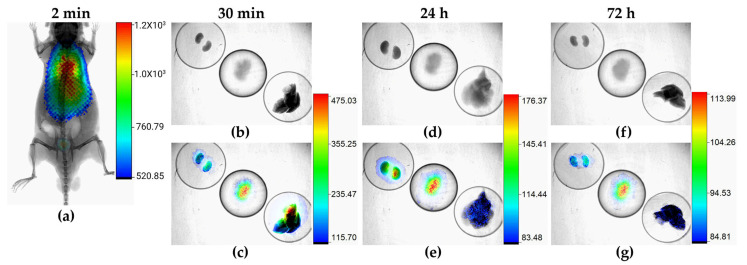
In vivo and ex vivo radioisotopic/X-ray images of athymic mice with lung micrometastases at different times after ^177^Lu-DOTA-PD-L1-i administration: (**a**) whole-body imaging; (**b**,**d**,**f**) X-ray images; (**c**,**e**,**g**) merged radioisotopic and X-ray images of kidneys (on the upper left side), lung (in the middle), and liver (on the lower right side). Note that the highest radioactivity accumulated in the lungs at all times examined (30 min, 24 and 72 h).

In vivo and ex vivo radioisotopic images of athymic mice with lung micrometastases after ^177^Lu-DOTA-PD-L1-i administration showed that the only tissue that accumulated the radiotracer was the lung invaded by HCC827 cancer cells, while the liver and kidneys captured and eliminated the radiotracer according to their physiological excretory function (Figure 11).

**Figure 12:**
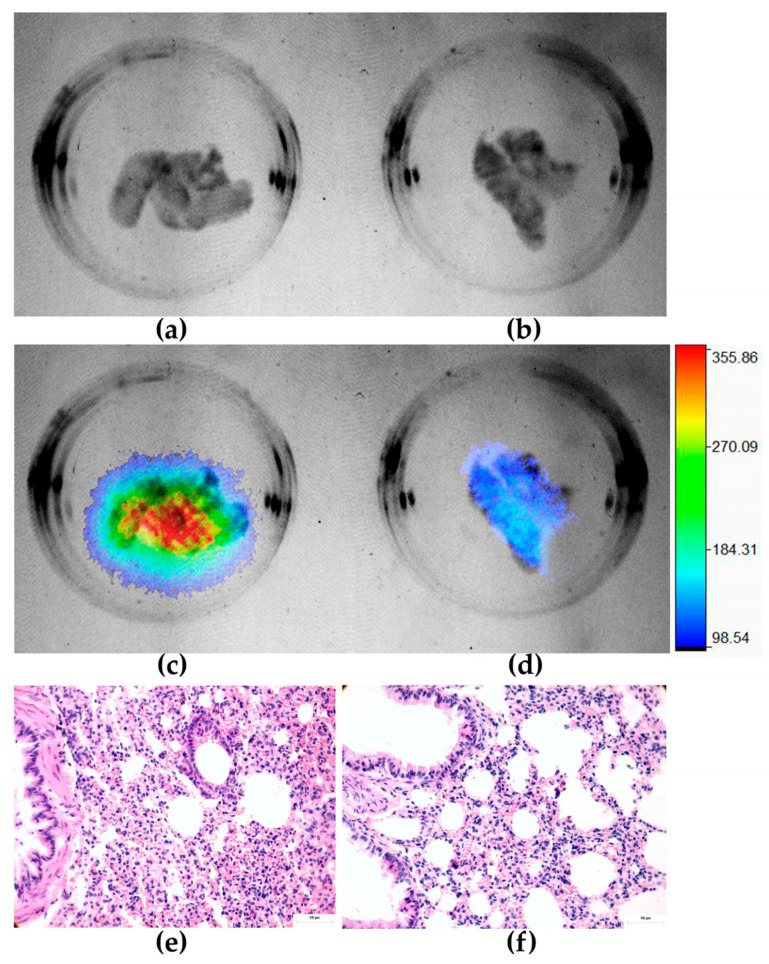
Ex vivo lungs from a mouse with lung micrometastases and injected with ^177^Lu-DOTA-PD-L1-i: (**a**) X-ray image; (**c**) merged radioisotopic/X-ray images; (**e**) lung parenchyma histological staining (H&E). Ex vivo lungs from an untreated mouse (control: without induced lung micrometastases) and injected with ^177^Lu-DOTA-PD-L1-i: (**b**) X-ray image; (**d**) merged radioisotopic/X-ray images; (**f**) lung parenchyma histological staining (H&E).

**Figure A4:**
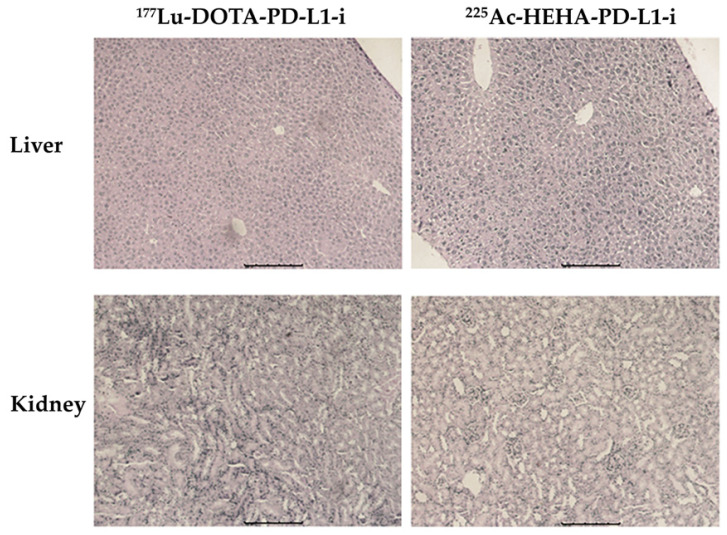
Microscopic views (×40) of kidney and liver tissues from mice exposed to ^177^Lu-DOTA-PD-L1-i and ^225^Ac-HEHA-PD-L1-i. Histological staining (H&E) analysis revealed no pathologic changes in the liver and kidney. There was no presence of any form of steatosis, cytoplasmic changes, or necrotic evidence. Hemorrhage and inflammatory infiltrations were absent. No evidence of cytotoxic degeneration was observed in nonparenchymal cells or hepatocytes.

In addition, paraffin sections of the lungs of mice injected with HCC827 cells were analyzed for growth within the lung parenchyma using hematoxylin and eosin histological staining (Figure 12e). Metastases were found in the lungs of all mice injected with cancer cells, as tumors grew within the lung parenchyma in the form of atypical nuclei and mitotic structures (Figure 12e). No metastases were found in the lungs of untreated control animals (control: without induced lung micrometastases) (Figure 12f). The metastatic adenocarcinoma tissue growing within the lung parenchyma distinctly differed from normal alveolar lung tissue and demonstrated biphasic growth of this cancerous tissue. Histological staining (H&E) analysis also revealed no pathologic changes in the liver and kidney of mice injected with the radiopharmaceuticals (Figure A4). There was no presence of any form of steatosis, cytoplasmic changes, or necrotic evidence. Hemorrhage and inflammatory infiltrations were absent (Figure A4). No evidence of cytotoxic degeneration was observed in nonparenchymal cells or hepatocytes (Figure A4). No significant cytotoxic effect was observed in the function of liver and kidney involved in metabolism, elimination, and or excretion of ^177^Lu-PD-L1-i and ^225^Ac-HEHA-PD-L1-i (Table A1).

The ^177^Lu-DOTA-PD-L1-i uptake pattern (average number of emitted photons per normalized area showed in the color bar of Figure 12) in the lungs of mice with metastatic HCC827 cells (298 ± 82 photons/cm^2^) was statistically significantly different (*p* < 0.05; Student’s *t*-test) compared with the group of mice in which metastases were not induced (78 ± 32 photons/cm^2^).

Immune checkpoint inhibitor (ICI) therapy using nanobodies, minibodies, antibodies, and, more recently, PD-L1 or PD-1 inhibitory peptides for nuclear molecular imaging have demonstrated their utility as highly specific biomolecules for targeting the tumor microenvironment (TME) [27]. Therefore, the radiotherapeutic PD-L1-i ligands of ^225^Ac and ^177^Lu developed in this research, with biokinetic and dosimetric properties suitable for delivering ablative radiation doses at the TME level, can potentially be combined with ICI immunotherapy and enhance the therapeutic effect in different types of cancer. It has been previously reported, at the preclinical and clinical levels, that radioimmunotherapy improves therapeutic outcomes, mitigates immune side effects, and reduces the induced radio-toxicity [36] in addition to promoting tumor regression of nonirradiated metastases in patients (abscopal effect), due to the power of radiation on increased cancer cell apoptosis and release of tumor-associated antigens with subsequent delivery of CD8+ T cells as an anti-tumor response [37]. However, poorly immunogenic tumors do not respond to ICI therapy. In these cases, the use of ^225^Ac-HEHA-PD-L1-i and ^177^Lu-DOTA-PD-L1-i in combination with the nonradiolabeled PD-L1-i ligand would be a viable option. This proposal is based on recent successful therapeutic results obtained by combining the radioligand ^177^Lu-PSMA-617 or ^225^Ac-PSMA-617 (targeting prostate cancer cells) with the pharmaceutical anti-PD-1 (pembrolizumab), which showed a marked synergistic effect and overcame immune limitations in patients with poorly immunogenic tumors [38].

The biodistribution of the radiopharmaceuticals analyzed in this study suggests that ^177^Lu-PD-L1-i may have a better biokinetic and dosimetric profile than ^225^Ac-PD-L1-i for use in targeted radiotherapy. Therefore, the results obtained justify that, as future work, preclinical studies of therapeutic efficacy should be carried out not only in a lung cancer model because, given the nature of PD-L1 gene expression in a wide range of cancer types, therapeutic efficacy should be evaluated in different models and cancer types induced and correlated with other markers of immune response.

## 3. Materials and Methods

Human plasma was provided by Alfa Aesar (USA). The macrocycles S-2-(4-isothiocyanatobenzyl)-1,4,7,10-tetraazacyclododecanic acid (DOTA-benzene-p-SCN) and 2-(4-isothiocyanatobenzyl)-1,2,7,10,13-hexaazacyclooctadecane-1,4,7,10,13,16-hexaacetic acid (HEHA-benzene-p-SCN) were obtained from Macrocyclics (Dallas, TX, USA). Lutetium-177 (^177^Lu), as ^177^LuCl_3_, was provided by ITM, Germany. Actinium-225 (^225^Ac), as ^225^AcCl_3_, was provided by IPPE Joint-Stock Company (Obninsk, Russia). HCC827 human lung cancer (PD-L1 positive) and C6 mouse glioma (PD-L1 negative) cell lines were purchased from ATCC^®^ (Manassas, VA, USA). All other reagents were purchased from Merk Millipore (Burlington, MA, USA). The PD-L1-I was synthesized, as previously reported [28], by a customized service, for research purposes, by Shanghai Yaxian Chemical Co., Ltd. (Jiading, Shanghai, China).

### 3.1. Synthesis and Chemical Characterization of DOTA-PD-L1-i and HEHA-PD-L1-i

DOTA-benzene-p-SCN (2 mg; 2.91 µmol) or HEHA-benzene-p-SCN (3 mg; 3.1 µmol) was dissolved in 1 mL of 0.2 M NaHCO_3_ buffer at pH 9.5, and then PD-L1-i ligand (6 mg; 3.2 µmol) was added. The mixture was incubated at 95 °C for 30 min. After cooling, 100 µL of ethanol was added. Finally, the conjugates were purified by preparative HPLC in line with a UV-vis detector (Waters) (Epic C18 Column, 5 μm, L:250 mm, Diam. Int: 20 mm, Perkin Elmer) using a linear gradient (rate flow of 4 mL/min) of CH_3_CN-0.1% TFA (B)/H_2_O-TFA (A), from 100% to 10% of A in 30 min. The fractions containing the conjugates (absorbance: 283 nm) were lyophilized.

Mass spectroscopy analysis (+Q1: 0.168 to 0.503 min, turbo spray, centroided) for the PD-L1-i peptide precursor was performed in an LC/MS system (Agilent 6460 series instrument) (Agilent Technologies, Santa Clara, CA, USA).

The IR spectra of DOTA-PD-L1-i and HEHA-PD-L1-i were acquired on an Agilent Technologies FT-IR 660 spectrometer (from 50 scans at 0.4 cm^−1^, from 400 to 4000 cm^−1^).

The UV-vis spectra (Thermo Fisher Genesys 10S spectrometer) of the PD-L1-i conjugates (1 mg/mL) were obtained from 210 to 300 nm.

### 3.2. Radiolabeling with ^177^Lu and ^225^Ac

DOTA-PD-L1-i or HEHA-PD-L1-i (1 mg) was dissolved in 50 µL of ethanol, adjusting to a final volume of 1 mL with 1 M acetate buffer, pH 5.0 to 20 µL of the PD-L1-i conjugates, 80 µL of ^177^LuCl_3_ (370 MBq in 0.01M HCl) or ^225^AcCl_3_ (370 kBq in 0.01M HCl) were added. Finally, the mixture was incubated at 95 °C for 60 min. After labeling, ^225^Ac-HEHA-PD-L1-I was purified using solid-phase extraction (Speak C18 cartridge, Waters). Radioactive solutions were diluted in injectable-grade water (1 mL) for further use. For comparative purposes, Lu-DOTA-PD-L1-i was also prepared under the same conditions using stable LuCl_3_ (anhydrous powder; 99.99% trace metal basis; Sigma-Aldrich: Saint Louis, MO, USA) followed by HPLC purification.

The radiochemical purity of the radioconjugates was determined by reversed-phase HPLC (Waters) with radiometric and UV-vis detectors. The analysis was carried out with a Discovery C18 column (particle size: 5 µm, length: 25 cm, diameter: 4.6 mm). A linear gradient (rate flow: 1 mL/min) of CH_3_CN-0.1% TFA (B)/H_2_O-0.1%TFA (A), from 100% to 10% of A in 30 min, was used. For ^225^Ac-HEHA-PD-L1-i, fractions of 1.0 mL were collected, and the activity was evaluated in a NaI(Tl) detector (Auto In-v-tron 4010; NML Inc., Houston, TX, USA).

### 3.3. Serum Stability

The ^225^Ac-HEHA-PD-L1-i and ^177^Lu-DOTA-PD-L1-i stability was evaluated in the medium they were obtained after labeling (water/ascorbic acid/sodium acetate) at 0.33, 3, 24, 48, and 72 h with radio-HPLC, as previously described. The conjugate solutions were diluted in human serum (5x) for stability assessment. The conjugates (n = 3) were incubated at 37 °C, and samples were taken at 0.33, 3, 24, 48, and 72 h.

### 3.4. Cellular Uptake

HCC827 human lung cancer (PD-L1 positive) and C6 mouse glioma (PD-L1 negative) cell lines were cultured in RPMI-1640 medium with penicillin and streptomycin (100 µg/mL) and fetal bovine serum (15%) in 5% CO_2_ at 37 °C. Cells diluted in PBS (pH 7) (1 × 10^5^ cells/tube) received two different treatments: (a) ^177^Lu-DOTA-PD-L1-i (4 kBq) (n = 3) and (b) ^225^Ac-HEHA-PD-L1-i (0.04 kBq) (n = 3). Cells were incubated with each treatment at 37 °C for 1 h. After incubation, tubes were measured in a NaI(Tl) detector to determine the initial activity (100%). The tubes were centrifuged at 500× *g* for 10 min. Then, a mixture of acetic acid/0.5 M NaCl was added, and the centrifugation was repeated. The liquid was removed, and the button activity was measured, corresponding to the percentage of activity captured by the cells regarding the initial activity.

### 3.5. Immunofluorescence

The HCC827 and C6 cell lines were fixed with 4% paraformaldehyde for 20 min, permeabilized with 0.5% TritonX-100, and 1% bovine serum albumin was used for cell blocking. Therefore, cells were incubated overnight with anti-PD-L1/CD274 antibody (FineTest Cat. FNab06280) at 1:25 or Cy7-conjugated PD-L1-i dilution prepared in agreement with the supplier instructions (Cy^®^7 Mono NHS Ester, pack of 1 mg, Merck Sigma-Aldrich). Cells incubated with anti-PD-L1/CD274 antibody were then incubated 1 h with Alexa Fluor 488-conjugated Goat anti-Rabbit IgG (H + L) (Invitrogen Cat. A32731). DAPI was used for intracellular fluorescence intensity observation for nuclei staining and fluorescence microscopy (Meiji Techno; Mod. MT6200: Saitama, Japan).

### 3.6. Cell Viability Assay

The effect of ^177^Lu DOTA-PD-L1-i, ^225^Ac-HEHA-PD-L1-i, and PD-L1-i on HCC827 cell viability (5 × 10^4^) was determined by flow cytometry with the Muse^®^ Count & Viability Kit from Luminex, DiaSorin, USA. Before taking readings with the Muse Cell Analyzer (Merck Millipore, Burlington, MA, USA) previously calibrated with the Muse^®^ System Check kit, the baseline detection was established using a fresh sample of untreated cells that served to delimit the size and granularity of the cell line, as well as the basal viability of the population. Also, the reading window for dead cells was established using an aliquot of the same population treated with 0.5% Triton-X100. Once the detection window for the HCC827 cells was established to discriminate between live and dead cells, 50 µL (2 × 10^4^ cells/200 µL) were taken from the cell suspension that had been treated with cold ligand (PD-L1-i, 0.1 µg), with 1.4 MBq of ^177^Lu DOTA-PD-L1-i or 0.37 kBq of ^225^Ac-HEHA-PD-L1-i for 5 h and then mixed with 450 µL of the viability reagent; after 5 min incubation at room temperature and in the dark, the samples were read in triplicate. For the calculation of the radiation absorbed dose to cell nuclei, the total number of nuclear transformations (N) normalized to the unit of administered activity (Bq^.^s/Bq) was obtained with the A(t) mathematical integration (from t = 0 to t = 5 h). Multiplying N by the dose factor (DF)(Gy/Bq.s) from the cytoplasm to the nucleus (MIRDcell software, version 2.1), the radiation absorbed dose was obtained. The progeny of ^225^Ac was considered. The model included cells with a diameter of 20 µm, a nucleus radius of 3 µm, and a density of 1 mg/mL.

### 3.7. Biodistribution and Studies

Under an approved protocol (No. 07-2018-2021), all animal procedures followed the ethical regulations for handling laboratory animals (NOM-062-ZOO-1999) and the Institutional Animal Care and Use Committee requirements. Male Nu/Nu (UPEAL, CINVESTAV, I.PN., Mexico City, Mexico) mice aged 6–8 weeks were maintained in a pathogen-free barrier facility. Mice were injected with 3.7 MBq (50 µL) of ^177^Lu DOTA-PD-L1-I or 3.7 kBq (50 µL) of ^225^Ac-HEHA-PD-L1-i in the tail vein and sacrificed at 0.5, 1, 3, 24, 72, and 96 h (n = 3). The heart, lungs, liver, pancreas, spleen, kidneys, small intestine, brain, and stomach were dissected to be measured in a NaI(Tl) radioactivity detector. The results are expressed as the percentage of injected dose (%ID) per organ. Blood samples were also extracted, and the activity was recorded as %ID/g. A second group of mice was inoculated intravenously with 1 × 10^6^ HCC827 cells. The animals were treated with 3.7 MBq (50 µL) of ^177^Lu DOTA-PD-L1-i (n = 3) or 3.7 kBq (50 µL) of ^225^Ac-HEHA-PD-L1-i (n = 3) via tail vein injection on day five post-tumor cell inoculation. The animals were dissected as described above. In the case of ^177^Lu DOTA-PD-L1-i, radioisotopic/X-ray in vivo/ex vivo images were obtained in a Preclinical Imaging System (Bruker, XTREME). For in vivo imaging, mice were anesthetized with oxygen and 2% isoflurane.

### 3.8. Absorbed Radiation Dose Assessment

The %ID values were used to obtain the biokinetic models ($q_{h}\left(t\right)$). The $A_{h}(t)$ functions were obtained by decay-correcting the biokinetic models, that is, by adding to the biological constant $(\lambda_{B}$) the radioactive constant $(\lambda_{R}$), as follows (Equation (1)):

(1) $$A\left(t\right)={Be}^{-(\lambda_{R}+\lambda_{B})t}+{Ce}^{-(\lambda_{R}+\lambda_{B})t}+{De}^{-(\lambda_{R}+\lambda_{B})t}$$

For dosimetry, ^225^Ac decay chain was considered. The biokinetic models’ integration (from t = 0 to t = ∞) calculated the N value in each murine organ. The DF values were those reported in the OLINDA 2.0 code.

### 3.9. Histopathological Assessment

Healthy lungs, kidneys, liver, and lungs from mice injected i.v. with HCC827 cells were fixed with 4% paraformaldehyde for 24 h at room temperature, embedded in paraffin, and sectioned in 4–5 µm thick sections using a microtome. Paraffin sections of lung samples were deparaffinized in xylene and rehydrated in a series of graded alcohols. Dewaxed tissue sections were stained with hematoxylin and eosin (H&E). The sections were assessed using a light microscope (magnification of 100x) (Zeiss; Mod. Axioscope: Oberkochen, Germany). Images were acquired with a digital camera (5 MP high-speed color; AmScope; Mod.Mu500: Irvine, CA, USA).

### 3.10. Evaluation of Creatinine and Liver Enzyme Levels

Mouse blood samples collected at 96 h after radiopharmaceutical administration were used for the determination of alanine aminotransferase (ALT), creatinine, and aspartate aminotransferase (AST). Creatinine was measured titrimetrically. The picrate method was used. AST and ALT were quantified using a Roche Diagnostics kit for UV assays.

## 4. Conclusions

The radiotherapeutic PD-L1-i ligands of ^225^Ac and ^177^Lu developed in this research, with biokinetic and dosimetric properties suitable for delivering ablative radiation doses at the tumor microenvironment level, could potentially be combined with immune checkpoint inhibitor therapy and enhance the therapeutic effect in various types of cancer. Considering the ease of preparation and high in vitro and in vivo stability, ^177^Lu-DOTA-PD-L1-i is proposed as the best option for in vivo PD-L1 protein-targeted radiotherapy.

## Figures and Tables

**Table 1:** Total nuclear transformations (N) and radiation absorbed dose in target organs of athymic mice produced by ^177^Lu-DOTA-PD-L1-i and ^225^Ac-HEHA-PD-L1-i.

Organ	^177^Lu-DOTA-PD-L1-i		^225^Ac-HEHA-PD-L1-i	
	$N=\int_{t=0}^{t=\infty }A\left(t\right)dt$ MBq·h	Radiation Absorbed Dose mGy/MBq	$N=\int_{t=0}^{t=\infty }A\left(t\right)dt$ MBq·h	Radiation Absorbed Dose mGy/Bq
Lung (HCC827 cancer cells)	59.3	43.0	18.9	5.15
Kidney	10.9	4.20	11.1	4.83
Liver	9.67	0.85	11.5	1.44

## Data Availability

Not applicable.

## Appendix B

**Table A1:** Alanine aminotransferase (ALT), creatinine, and aspartate aminotransferase (AST) in mice after 96 h administration of 3.7 MBq of ^177^Lu DOTA-PD-L1-i (n = 3) or 3.7 kBq of ^225^Ac-HEHA-PD-L1-i (n = 3) on day 5 after intravenous inoculation of HCC827 lung cancer cells (mean ± standard deviation). No significant differences (*p* < 0.05) in the ALT, creatinine, and AST values between mice treated with radiopharmaceuticals and those of the control group were found.

Mice Group	Alanine Aminotransferase (ALT) (IU/L)	Creatinine (mg/dL)	Aspartate Aminotransferase (AST) (IU/L)
^177^Lu DOTA-PD-L1-i	68 ± 4	0.199 ± 0.062	143 ± 14
^225^Ac-HEHA-PD-L1-i	72 ± 5	0.202 ± 0.053	147 ± 18
Control	71 ± 7	0.198 ± 0.057	150 ± 13

## References

[1] Arneth B. (2019). Tumor microenvironment. Medicina.

[2] Charpentier M., Spada S., Van Nest S.J., Demaria S. (2022). Radiation therapy-induced remodeling of the tumor immune microenvironment. Semin. Cancer Biol..

[3] Musetti S., Huang L. (2018). Nanoparticle-mediated remodeling of the tumor microenvironment to enhance immunotherapy. ACS Nano.

[4] Jailkhani N., Ingram J.R., Rashidian M., Rickelt S., Tian C., Mak H., Jiang Z., Ploegh H.L., Hynes R.O. (2019). Noninvasive imaging of tumor progression, metastasis, and fibrosis using a nanobody targeting the extracellular matrix. Proc. Natl. Acad. Sci. USA.

[5] Demmer O., Gourni E., Schumacher U., Kessler H., Wester H.J. (2011). PET imaging of CXCR4 receptors in cancer by a new optimized ligand. ChemMedChem.

[6] Ávila-Sánchez M., Ferro-Flores G., Jiménez-Mancilla N., Ocampo-García B., Bravo-Villegas G., Luna-Gutiérrez M., Santos-Cuevas C., Azorín-Vega E., Aranda-Lara L., Isaac-Olivé K. (2020). Synthesis and preclinical evaluation of the 99m Tc-/177 Lu-CXCR4-L theranostic pair for in vivo chemokine-4 receptor-specific targeting. J. Radioanal. Nucl. Chem..

[7] Mikaeili A., Erfani M., Shafiei M., Kobarfard F., Abdi K., Sabzevari O. (2018). Development of a 99mTc-labeled CXCR4 antagonist derivative as a new tumor radiotracer. Cancer Biother. Radiopharm..

[8] Lindner T., Loktev A., Altmann A., Giesel F., Kratochwil C., Debus J., Jäger D., Mier W., Haberkorn U. (2018). Development of quinoline-based theranostic ligands for the targeting of fibroblast activation protein. J. Nucl. Med..

[9] Trujillo-Benítez D., Luna-Gutiérrez M., Ferro-Flores G., Ocampo-García B., Santos-Cuevas C., Bravo-Villegas G., Morales-Ávila E., Cruz-Nova P., Díaz-Nieto L., García-Quiroz J. (2022). Design, Synthesis and Preclinical Assessment of 99mTc-iFAP for In Vivo Fibroblast Activation Protein (FAP) Imaging. Molecules.

[10] Loktev A., Lindner T., Burger E.-M., Altmann A., Giesel F., Kratochwil C., Debus J., Marmé F., Jäger D., Mier W. (2019). Development of fibroblast activation protein–targeted radiotracers with improved tumor retention. J. Nucl. Med..

[11] Vallejo-Armenta P., Ferro-Flores G., Santos-Cuevas C., García-Pérez F.O., Casanova-Triviño P., Sandoval-Bonilla B., Ocampo-García B., Azorín-Vega E., Luna-Gutiérrez M. (2022). [99mTc] Tc-iFAP/SPECT Tumor Stroma Imaging: Acquisition and Analysis of Clinical Images in Six Different Cancer Entities. Pharmaceuticals.

[12] Dabir M., Novruzov E., Mattes-György K., Beu M., Dendl K., Antke C., Koerber S., Röhrich M., Kratochwil C., Debus J. (2023). Distinguishing benign and malignant findings on [68 ga]-FAPI PET/CT based on quantitative SUV measurements. Mol. Imaging Biol..

[13] Ludwig B.S., Kessler H., Kossatz S., Reuning U. (2021). RGD-binding integrins revisited: How recently discovered functions and novel synthetic ligands (re-) shape an ever-evolving field. Cancers.

[14] Van de Wiele C., Sathekge M., De Spiegeleer B., De Jonghe P.J., Debruyne P.R., Borms M., Beels L., Maes A. (2020). PSMA expression on neovasculature of solid tumors. Histol. Histopathol..

[15] An S., Huang G., Liu J., Wei W. (2022). PSMA-targeted theranostics of solid tumors: Applications beyond prostate cancers. Eur. J. Nucl. Med. Mol. Imaging.

[16] Hofstetter G., Grech C., Pils D., Pammer J., Neudert B., Pötsch N., Baltzer P., Traub-Weidinger T., Seebacher V., Aust S. (2022). Prostate-Specific Membrane Antigen (PSMA) Expression in Tumor-Associated Neovasculature Is an Independent Prognostic Marker in Patients with Ovarian Cancer. J. Pers. Med..

[17] Vallejo-Armenta P., Soto-Andonaegui J., Villanueva-Pérez R.M., González-Díaz J.I., Contreras-Contreras K., Bautista-Wong C.G., Sandoval-Bonilla B., Nettel-Rueda B., Santos-Cuevas C., Ferro-Flores G. (2021). [99mTc] Tc-iPSMA SPECT brain imaging as a potential specific diagnosis of metastatic brain tumors and high-grade gliomas. Nucl. Med. Biol..

[18] Hernández-Jiménez T., Cruz-Nova P., Ancira-Cortez A., Gibbens-Bandala B., Lara-Almazán N., Ocampo-García B., Santos-Cuevas C., Morales-Avila E., Ferro-Flores G. (2022). Toxicity Assessment of [177Lu]Lu−iFAP/iPSMA Nanoparticles Prepared under GMP-Compliant Radiopharmaceutical Processes. Nanomaterials.

[19] Luna-Gutiérrez M., Ocampo-García B., Jiménez-Mancilla N., Ancira-Cortez A., Trujillo-Benítez D., Hernández-Jiménez T., Ramírez-Nava G., Hernández-Ramírez R., Santos-Cuevas C., Ferro-Flores G. (2022). Targeted Endoradiotherapy with Lu_2_O_3_-iPSMA/-iFAP Nanoparticles Activated by Neutron Irradiation: Preclinical Evaluation and First Patient Image. Pharmaceutics.

[20] Ocampo-García B., Lara L.A., Ferro-Flores G., Morales-Avila E., Isaac-Olivé K. (2022). Role of Nanotechnology in Biological Therapies. Nanomater. Nanotechnol. Med..

[21] Melendez-Alafort L., Ferro-Flores G., De Nardo L., Ocampo-García B., Bolzati C. (2023). Zirconium immune-complexes for PET molecular imaging: Current status and prospects. Coord. Chem. Rev..

[22] Hamid O., Robert C., Daud A., Hodi F.S., Hwu W.-J., Kefford R., Wolchok J.D., Hersey P., Joseph R.W., Weber J.S. (2013). Safety and tumor responses with lambrolizumab (anti–PD-1) in melanoma. N. Engl. J. Med..

[23] Garassino M.C., Gadgeel S., Speranza G., Felip E., Esteban E., Dómine M., Hochmair M.J., Powell S.F., Bischoff H.G., Peled N. (2023). Pembrolizumab plus pemetrexed and platinum in nonsquamous non–small-cell lung cancer: 5-year outcomes from the phase 3 KEYNOTE-189 study. J. Clin. Oncol..

[24] Chatterjee S., Lesniak W.G., Miller M.S., Lisok A., Sikorska E., Wharram B., Kumar D., Gabrielson M., Pomper M.G., Gabelli S.B. (2017). Rapid PD-L1 detection in tumors with PET using a highly specific peptide. Biochem. Biophys. Res. Commun..

[25] De Silva R.A., Kumar D., Lisok A., Chatterjee S., Wharram B., Venkateswara Rao K., Mease R., Dannals R.F., Pomper M.G., Nimmagadda S. (2018). Peptide-based 68Ga-PET radiotracer for imaging PD-L1 expression in cancer. Mol. Pharm..

[26] Zhou X., Jiang J., Yang X., Liu T., Ding J., Nimmagadda S., Pomper M.G., Zhu H., Zhao J., Yang Z. (2022). First-in-humans evaluation of a PD-L1–binding peptide PET radiotracer in non–small cell lung cancer patients. J. Nucl. Med..

[27] Krutzek F., Kopka K., Stadlbauer S. (2022). Development of Radiotracers for Imaging of the PD-1/PD-L1 Axis. Pharmaceuticals.

[28] Miller M.M., Mapelli C., Allen M.P., Bowsher M.S., Boy K.M., Gillis E.P., Langley D.R., Mull E., Poirier M.A., Sanghvi N. (2016). Macrocyclic Inhibitors of the pd-1/pd-l1 and cd80 (b7-1)/pd-l1 Protein/Protein Interactions. Bristol Myers Squibb Co. U. S. A. 20161093pp.

[29] Ferro-Flores G., Arteaga de Murphy C., Melendez-Alafort L. (2006). Third generation radiopharmaceuticals for imaging and targeted therapy. Curr. Pharm. Anal..

[30] Lacoeuille F., Arlicot N., Faivre-Chauvet A. (2018). Targeted alpha and beta radiotherapy: An overview of radiopharmaceutical and clinical aspects. Médecine Nucléaire.

[31] Deal K.A., Davis I.A., Mirzadeh S., Kennel S.J., Brechbiel M.W. (1999). Improved in vivo stability of actinium-225 macrocyclic complexes. J. Med. Chem..

[32] Thiele N.A., Wilson J.J. (2018). Actinium-225 for Targeted a Therapy: Coordination Chemistry and Current Chelation Approaches. Cancer Biother. Radiopharm..

[33] Hernández-Jiménez T., Ferro-Flores G., Morales-Ávila E., Isaac-Olivé K., Ocampo-García B., Aranda-Lara L., Santos-Cuevas C., Luna-Gutiérrez M., De Nardo L., Rosato A. (2022). 225Ac-rHDL Nanoparticles: A Potential Agent for Targeted Alpha-Particle Therapy of Tumors Overexpressing SR-BI Proteins. Molecules.

[34] Bannik K., Madas B., Jarzombek M., Sutter A., Siemeister G., Mumberg D., Zitzmann-Kolbe S. (2019). Radiobiological effects of the alpha emitter Ra-223 on tumor cells. Sci. Rep..

[35] Manohar S., Kompotiatis P., Halfdanarson T.R., Hobday T.J., Thorpe M., Johnson G.B., Kendi A.T., Leung N. (2021). 177Lu-dotatate use in chronic kidney disease patients: A single center experience. J. Onco-Nephrol..

[36] Li H., Luo Q., Zhang H., Ma X., Gu Z., Gong Q., Luo K. (2023). Nanomedicine embraces cancer radio-immunotherapy: Mechanism, design, recent advances, and clinical translation. Chem. Soc. Rev..

[37] Abuodeh Y., Venkat P., Kim S. (2016). Systematic review of case reports on the abscopal effect. Curr. Probl. Cancer.

[38] Bellavia M.C., Patel R.B., Anderson C.J. (2022). Combined Targeted Radiopharmaceutical Therapy and Immune Checkpoint Blockade: From Preclinical Advances to the Clinic. J. Nucl. Med..

